# Characterization of the complete chloroplast genome of *Pinus yunnanensis* var. *pygmaea*

**DOI:** 10.1080/23802359.2020.1800433

**Published:** 2020-07-31

**Authors:** Tian Hong Hong, Zhao Yang, Wang Xiu-Rong, Xiao Feng, Yayan Zhu

**Affiliations:** a College of Forestry, Guizhou University, Guiyang, PR China; b Institute for Forest Resources & Environment of Guizhou, Guizhou University, Guiyang, PR China; c Key Laboratory of Forest Cultivation in Plateau Mountain of Guizhou Province, Guizhou University, Guiyang, PR China; d Key Laboratory of Plant Resource Conservation and Germplasm Innovation in Mountainous Region (Ministry of Education), Guizhou University, Guiyang, PR China; e Guizhou Academy of Forsetry, Guiyang, PR China

**Keywords:** *Pinus yunnanensis* var. *pygmaea*, high throughput sequencing, chloroplast genome

## Abstract

*Pinus Yunnanensis* var. *pygmaea* (Fam: pinaceae; Gen: *Pinus*), is a mutant of *Pinus yunnanensis*. Franch. To contribute to its conservation, the complete chloroplast (cp) genome of *P. yunnanensis* var. *Pygmaea* was sequenced and assembled by high-throughput sequencing technology. The results show that *P. yunnanensis* var. *pygmaea cp* genome contained 101 genes, including 64 protein-coding genes, 33 *tRNA* genes, and four *rRNA* genes. The overall GC content of the *cp* genome is 38.50%. A phylogenetic tree reconstructed by 16 *cp* genomes reveals that *P. yunnanensis* var. *pygmaea* is most related with *Pinus taiwanensis*.

*Pinus Yunnanensis* var. *pygmaea* (Fam: pinaceae; Gen: *Pinus*), is a mutant of *Pinus yunnanensis*. Franch., distributed in Muli and Zhaojue in southwestern Sichuan Province and the mountains of northwestern and central Yunnan Province, China. The *P. pygmaea* (*P. yunnanensis* var. *pygmaea*) is inconspicuous, and the basal part has many trunks, which are clustered, cones are clustered, which are persistent on the tree after ripening; the fruiting phenomenon is generally early (Zheng-li et al. 1994; Yu-Lan et al. 2012).

At present, cp genome, mitochondrial genome, and nucleus genome are three major genomes used for phylogenetic studies in plant, among which cp genome is widely used in the study of kinship between different species and species evolution because of its conserved number and sequence of genes (Njuguna et al. 2013).

The *P. pygmaea* seeds were collected from Kunming City, Yunnan Province, China (E:103°13′; N:24°52′), and kept at the Institute of Forest Resources & Environment Center of Guizhou Province, the deposit number is NO. PY-001-1. Collected annual new needles for Illumina NovaSeq sequencing to obtain the cp genome. The *P. pygmaea* cp genome sequence was obtained by SPAdes (Bankevich et al. 2012) and A5-miseq (Coil et al. 2015) splicing fragments. The annotated cp genome sequence has been deposited into the Genbank (accession: MT712078).

*P. pygmaea* cp genome was not distinctly tetragonal with 119,570 bp in length. The content of GC is 38.50%, the annotated complete cp genome contains 101 genes, including 64 protein-coding gene, four ribosomal RNA, and 33 transfer RNA.

The phylogenetic relationships of 16 different species of the *P. pygmaea* were investigated using the evolutionary tree construction method using IQ-tree software (Nguyen et al. 2015) with maximum-likelihood (ML) method. A phylogenetic tree reconstructed by 16 cp genomes reveals that *P. pygmaea* is most related with *Pinus taiwanensis* (Figure 1). The results of the phylogenetic relationships are consistent with the results of the morphological classification, laying the foundation for the study of the evolution of pinaceae species.

**Figure 1.**
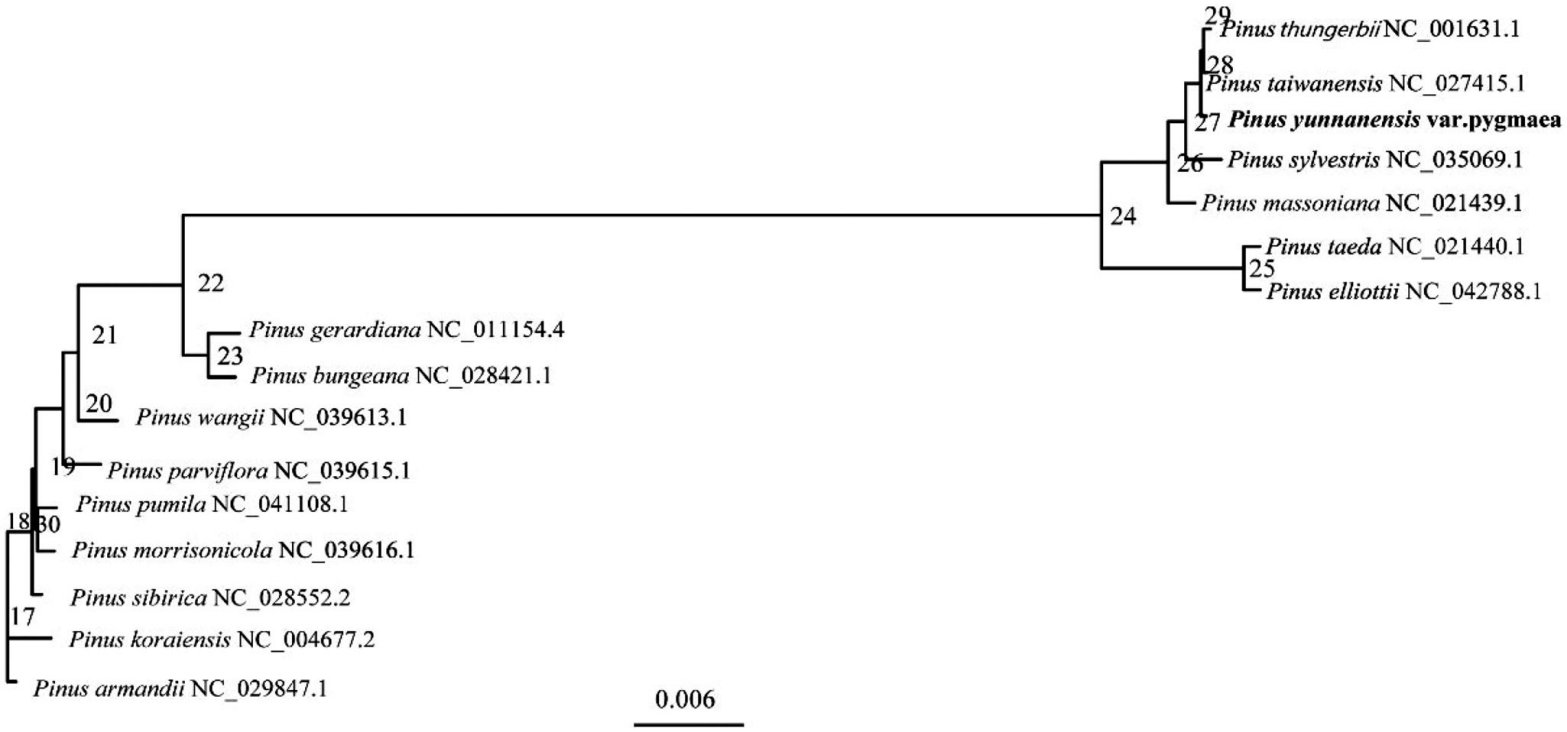
Phylogenetic relationships of 16 species based on the maximum-likelihood (ML) analysis of *P. pygmaea*.

## Data Availability

The data that support the findings of this study are openly available in GenBank of NCBI at https://www.ncbi.nlm.nih.gov, reference number MT712078.
